# Surgery

**Published:** 1895-12

**Authors:** 


### SURGERY.

EDITED BY FLOYD W. MCRAE, M.D.

### SOME RECENT VIEWS ON APPENDICITIS.*

1. The explanation of the great frequency of inflammation of the appendix is to be found in the following facts:

(*a*) It is a functionless structure of low vitality, removed from the direct fecal current; it has a scanty mesentery so attached to both cecum and ileum, that it is easily stretched or twisted when they become distended; it derives its blood supply through a single vessel, the caliber of which is seriously interfered with or altogether occluded by anything which produces dragging upon the mesentery.

(*b*) In addition, there is almost always present a micro-organism—the *bacterium coli commune*—capable of great virulence when there is constriction of the appendix or lesions of its mucous coat or of its parietes.

2. The symptoms in a case of mild catarrhal appendicitis—general abdominal pain, umbilical pain, localized pain and tenderness

*Conclusions of an address delivered by J. William White, M.D., Professor of Clinical Surgery, University of Pennsylvania, before the Surgical Section of the College of Physicians of Philadelphia.

on pressure in the right iliac fossa, vomiting, moderate fever, and slightly increased pulse-rate—cannot at present with any certainty be distinguished from the symptoms, apparently precisely identical, which mark the onset of a case destined to be of the very gravest type.

3. It must be determined by future experience whether or not operation in every case of appendicitis, as soon as the diagnosis is made, would be attended by a lower mortality than would waiting for more definite symptoms indicating unmistakably the need of operative interference. At present such indication exists in every case if the onset is sudden and the symptoms markedly severe, and whenever in a mild case the symptoms are unrelieved at the end of forty-eight hours, or, *a fortiori*, if at that time they are growing worse.

4. It must be determined by future experience whether cases seen from the third to the sixth day, which present indications of the beginning circumscription of the disease by adhesions, and which tend to the formation of localized abscesses, will do better with immediate operation, with the risk of infecting the general peritoneal cavity, or with later operation when the circumscribing wall is stronger and less likely to be broken through. At present, operation is certainly indicated whenever a firm, slowly-forming, well-defined mass in the right iliac fossa is to be felt; or, on the other hand, when a sudden increase in the sharpness and the diffusion of the pain and tenderness points to perforation of the appendix or breaking down of the limiting adhesions.

5. In the beginning of general suppurative peritonitis, operation offers some hope of success. In the presence of general peritonitis with septic paresis of the intestines, operation has thus far been useless.

6. Recurrent appendicitis of mild type, like acute appendicitis, frequently results from digestive derangements. Several attacks may occur followed by entire and permanent recovery, but it is as yet impossible to differentiate these cases accurately from those which do not tend to spontaneous cure. Operation is certainly indicated whenever the attacks are very frequent.

7. Chronic relapsing appendicitis is characterized by the persistence of local symptoms during the intervals, and by more or less failure of the general health. It usually indicates operation.

8. In either the recurrent or the chronic relapsing variety, operation should be advised according to the following indications formulated by Treves: whenever (1) The attacks have been very numerous. (2) The attacks are increasing in frequency and severity. (3) The last attack has been so severe as to place the patient's life in considerable danger. (4) The constant relapses have reduced the patient to the condition of a chronic invalid, and have rendered him unfit to follow any occupation. (5) Owing to the persistence of certain local symptoms during the quiescent period, there is a probability that a collection of pus exists in or about the appendix.—*College and Clinical Record.*

---

### RAILWAY INJURIES.

A late *Railway Surgeon* contains an interesting analysis of 24,994 injuries—of this number 21,128 were inflicted upon employees, 984 upon passengers, 2,529 upon other persons. As regards the character of employment most dangerous to the railway employees, we will only consider those directly concerning the transportation department. We find that out of the 21,128 employees injured, 3,888 were brakemen, 1,504 switchmen, 1,323 firemen, 698 engineers. It will thus be seen that the brakeman is the most frequently injured individual upon the railway; next to him comes the yardman, with less than one-half of the number of injuries. It will likewise be noticed that about half as many engineers get hurt as firemen, which, of course, can be readily explained from the fact that as a general thing the fireman's injuries are more trivial than the engineer's, and aside from this fact the fireman is constantly passing from the engine to the tender and places himself in the power of gravity, and is thus more frequently hurt.

As regards the manner of injury, we find that owing to the great inequality of couplers and the dangerous positions assumed in handling the cars, that we have 3,686 injuries occurring by coupling alone, while next in order follow jumping off trains in motion, the injuries due to this cause numbering 1,023; while from falling off trains in motion the injuries number 901. The jolt, the jar, the play of gravity, uncertain footing, and probably intemperance, influence this. We find that 672 were injured by derailments; 571 by collisions; 538 by attempting to board moving trains. As regards passengers, 211 were injured in derailments; 193 in collisions, and 82 fell from moving trains. Under this last head, those placed under the heading of boarding moving trains are included.

Regarding non-employees we find that they were injured in the following manner: 788 while walking on the track; 353 in attempting to board moving trains; and 316 by falling and jumping from moving trains; doubtless some of them were thrown off. One hundred and seventy-seven were injured by being drunk on the track, and 88 by sleeping on the track; 8 of them willfully killed themselves by throwing themselves in front of moving trains.

It will not be uninteresting to specify the character of the injuries occurring to the 21,128 employees, as regards the part of the anatomy which suffered most: The upper extremity sustained 9,428 injuries, plainly for the reason of the broader use of this extremity. The lower extremity sustained 6,434 injuries. The head and face receiving 2,652, while the trunk or body only sustained 1,551 injuries. We find that sprains are very frequent in railway service, simply for the reason of excessive muscular use and great stress applied under certain conditions. Thus we have 3,213 sprains produced upon various portions of the body. The ankle joint being the most frequent joint in the body to suffer from sprains. One thousand three hundred and fifty-nine sprains occurred to this joint. We find that dislocations prevail with the upper extremity—155 occurred to this portion of the body while only 36 involved the lower extremity. The shoulder joint is the most frequent joint in the body to suffer dislocation, the next in order of the upper extremities is the elbow joint, while the knee joint in the lower extremity suffers more from dislocation than any other.

The tibia and fibula are more frequently fractured than any other bones of the body, except those of the hand. The clavicle certainly does not keep up its reputation for frequency of fracture in comparison to the bones of the inferior extremity as mentioned.

### THE OPERABILITY OF CANCER.

Roux, Lausanne. (*Med. Week*, III, No. 19, p. 219.)

Formerly the theory of constitutional cancer, the lack of antiseptic measures, and the fear of recurrence were enough to discourage even a daring surgeon from attempting more than the most restricted and palliative operations in cases of maligant tumors. The characteristic features of cancer tend to show that it is a parasitic disease, resembling tuberculosis in its clinical evolution. Cancer should, therefore, be attacked with as much promptness and energy as the latter affection. The trouble frequently is that we operate too late, when recurrence is unavoidable, and this is the reason why older statistics are so very unfavorable, and recent ones are far from encouraging. The moment the diagnosis is certain, operation should be resorted to, taking care, under all circumstances and in all situations, to carefully look after the glands in the neighborhood, even if they are apparently in a healthy condition.

That delay in intervention is the principal cause of recurrence is evident from personal observation of three groups of cancers, viz., those of the mamma, uterus (except the appendages), and gastro-intestinal tract.

In cancer of the stomach, 12 per cent. of all cases were operable, 88 per cent. inoperable, a part of the latter being amenable to a palliative operation.

The death-rate in pylorectomy for the relief of cancer was from 14 to 20 per cent., that of gastro-enterostomy 14 per cent.

In cancer of the uterus, 68 per cent. of all cases were inoperable and 32 per cent. operable; only a third of the latter could be operated upon through the sacrum. These would have been inoperable six years ago!

The death-rate in vaginal hysterectomy was 8, and in sacral hysterectomy 11 per cent.

In cancer of the mamma, a condition in which operation is most frequently resorted to, 12 per cent. of all cases were absolutely inoperable, and in 50 per cent. a speedy recurrence could be foreseen. The proportion of deaths from operation was 5 per cent., which is the same as the general death-rate in all kinds of operations.

There is no doubt that early intervention would be apt to prevent recurrence, seeing that even now, when the conditions are anything but favorable, recoveries are obtained. He has operated in cases of gastro-intestinal cancer, in which three years have now elapsed without recurrence, and in cancers of the rectum recovery has been maintained for four years; the same holds good of a cancer of the uterus with involvement of the wall of the bladder; and a villous cancer of the kidney, in which the operation, performed five years ago, proved to be an extremely difficult one, has not recurred. He has extirpated the tongue in a case of recurrence nine years after an operation performed by Professor Kocher. In a case of cancerous goitre, the patient has survived operation eleven years; and in another case of operation for cancer of the testicle, no recurrence has taken place for twelve years. In view of the results of histological examination, no doubt can be entertained as to the true nature of these tumors.—*American Medical and Surgical Bulletin.*

---

### SUPRAPUBIC OPENING OF THE BLADDER.

Pousson (*Bulletin et Mémoires de la Société de Chirurgie*, Tome xx, 1894, p. 712) points out the different indications for suprapubic cystotomy and cystostomy. The latter name is applied to the operation of Poncet, in which the mucous membrane of the bladder is sutured to the skin. Physiologically, the cystostomy opening acts as a simple fistula, leaving the urine to flow incessantly. The cystostomy opening acts as a canal with supple walls, if not actually contractile, applied one to the other, and permitting the accumulation of a certain amount of urine which can be expelled voluntarily. The indications for cystotomy are clear; it is the ultimate resort of the surgeon in the presence of a vesical neuralgia, of a painfu cystitis, and of a persistent hemorrhage. The author performed it six times—four for painful cystitis, one for cystitis with hemorrhage, and one for hemorrhage due to a tumor. In four of these six cases the patients were relieved of their pains and hemorrhage. The indications for cystostomy are less clear. Poncet advised it for prostatic troubles. The author relies on catheterism. He has resected the prostate in two cases, one of which recovered, but only resorted to cystostomy in a single case of perineal fistula. The operation, while it may be valuable in a small number of prostatic patients, will find its field of usefulness principally in other affections of the urethra.

In a discussion on the same subject, immediately following the paper by Pousson (page 714), Routier gives his experience. He operated by cystostomy five times for painful cystitis with one death. The fatal case was one of tuberculosis; one of the others was a blennorrhagic cystitis in a young man aged twenty years, and another was in a woman who progressed unfavorably, but who recovered by means of a subsequent vesico-vaginal opening.

For chronic troubles he operated seven times with three deaths. In those patients who recover from the operation the prostate shrinks and they again urinate by urethra. They can retain considerable urine in the bladder.

Tuffier (page 721) said that the discussion had only brought out twelve cases in which cystostomy had been done for prostatic obstruction; this showed that the indications for it are very rare. The points decided are that the acute retention in enlargement of the prostate is to be treated by catheterism or leaving a catheter in the bladder (*sonde à demeure*); that when this course is impossible, and infection is present, and repeated punctures insufficient, then cystostomy is indicated, with the object of abandoning it as soon as the prostatic congestion disappears and the prostate lessens in size, as is the rule, and resuming treatment by the urethra. Frequent micturition alone is not an indication, but persistent pain and hemorrhages are. In certain cases vesical infection is also an indication.—*University Medical Magazine.*

---

### REPORT OF NINE HUNDRED EXTIRPATIONS OF GOITRE.

In commenting on his "Report of Extirpation of Goitre" (*Journal Nervous and Mental Diseases*), Kocher remarked, during the past ten years, in which he had performed the operation on 900 patients, he had met but one case in which cachexia strumipriva had developed. This was entirely owing to the fact that he always left a part of the thyroid gland, which was sufficient to carry on the functions of that organ. In the single case in which the cachexia developed, the extirpation was unilateral, but after the operation it was found that the other side was trophied.

A very interesting statement by Kocher is that he had several times noticed that tetany, which he is pleased to call acute cachexia, developed. It is interesting, likewise, to hear that the patient in whom cachexia strumipriva developed recovered through thyroid feeding.

In speaking of the mortality in his operations, Kocher says that he deducts thirty cases of malignant goitres in which unusual and peculiar difficulties militated against success. Of the remaining 870, eleven died; but in six only was death the direct result of the operation, and of these, three were operated on for Graves's disease. The extirpation of the goitre in the latter disease he regards as dangerous. For the latter he prefers to ligature the thyroid arteries; but never more than three of them.

Referring to some researches that had been made under his observation by Lanz and Trachewski in the treatment of goitre by the injection of thyroid extract, the author said that in the long run this method may determine complete atrophy of healthy parts of the thyroid gland. He further remarked that all the symptoms of Graves's disease had been produced in healthy animals by these experiments. He found also that the symptoms of exophthalmic goitre improved greatly under treatment by phosphate of sodium.—*Maryland Medical Journal.*

---

### CIRCULAR SUTURE OF THE INTESTINE.

Bier reports from Esmarch's clinic fifteen instances of suture of the intestine in fourteen patients, with only two deaths, and in neither of these was the suture responsible. Such excellent results give him authority to speak, and he uses his influence in favor of the simplest method for circular suture of the intestine, namely, a single row of interrupted fine-silk Lembert sutures. His technique is simple, and does not differ from that in common use, except that he objects to the employment of very fine needles and silk, preferring both of a somewhat larger size, because more easily handled. He claims to make his suture in fifteen or twenty minutes, which indicates great dexterity in the operator.—*American Medical and Surgical Bulletin.*

